# Association Between Admission Estimated Glomerular Filtration Rate and Long-Term Mortality in Patients with Acute Myocardial Infarction

**DOI:** 10.3390/jcm15208096

**Published:** 2026-10-19

**Authors:** Guy Vishnevsky, Ammie Wolf, Yoav Perez, Shir Frydman, Shafik Khoury, Reem Elbassel, Yan Topilsky, Yacov Shacham

**Affiliations:** 1 Department of Cardiology, Tel Aviv Sourasky University Medical Center, Tel Aviv 6423906, Israel; 2 Gray Faculty of Medical & Health Sciences, Tel Aviv University, Tel Aviv 6997801, Israel; 3 Internal Medicine H, Tel Aviv Sourasky University Medical Center, Tel Aviv 6423906, Israel

**Keywords:** eGFR, acute kidney injury (AKI), ST-segment elevation myocardial infarction (STEMI), chronic kidney disease (CKD)

## Abstract

**Background**: Reduced estimated glomerular filtration rate (eGFR) and acute kidney injury (AKI) are common in patients with ST-segment elevation myocardial infarction (STEMI) and adversely influence prognosis. This retrospective cohort study evaluated the association between admission eGFR and one-year mortality following STEMI, accounting for admission AKI and cardiovascular risk factors. **Methods**: Consecutive adults (n = 2903) admitted with STEMI undergoing primary percutaneous coronary intervention (PCI) at a tertiary center were analyzed, selected from 2949 initially screened patients after excluding those treated conservatively or with thrombolysis (n = 14), those with a final discharge diagnosis other than STEMI (n = 21), and early deaths within 24 h of presentation (n = 11). Admission eGFR was calculated using the 2021 CKD-EPI creatinine equation and categorized into quintiles. The primary outcome was all-cause one-year mortality, assessed using multivariable logistic regression and secondary Kaplan–Meier survival analysis. **Results**: Both one-year and 30-day crude mortality decreased markedly across increasing eGFR quintiles (*p* < 0.001), a pattern that was maintained among patients presenting without admission AKI (30-day mortality: Q5 4.4% vs. Q1 0.0%, *p* < 0.001). In crude analysis, 30-day mortality was 9.1% in the lowest eGFR quintile overall. Multivariable logistic regression identified advancing age (OR 1.05 per year, *p* < 0.001) and admission AKI (OR 7.07, *p* < 0.001) as the dominant independent predictors of one-year mortality, whereas individual eGFR quintiles were attenuated after adjustment (global Wald *p* = 0.047). **Conclusions**: Lower admission eGFR is significantly associated with one-year mortality after STEMI in crude and unadjusted analyses, but its predictive contribution is substantially attenuated following multivariable adjustment for advancing age and admission AKI, which serve as the dominant prognostic determinants. Rather than acting as a strong isolated independent predictor, admission renal function primarily serves as an indicator of baseline cardiorenal reserve and baseline patient vulnerability.

## 1. Introduction

ST-segment elevation myocardial infarction (STEMI) continues to be a major cause of morbidity and mortality despite major advances in percutaneous coronary interventions (PCI) and guideline-directed pharmacologic management strategies. The bidirectional crosstalk between the cardiovascular and renal systems—broadly conceptualized as cardiorenal syndrome—creates a vulnerable pathophysiological state that significantly complicates acute clinical management [1,2]. A significant proportion of patients with STEMI present with acute kidney injury (AKI) or develop AKI during hospitalization, which is associated with worse clinical outcomes and increased short- and long-term mortality [3,4]. Regardless of AKI, admission renal function is well known to be associated with STEMI and other acute coronary syndrome (ACS) outcomes [5,6,7].

Glomerular filtration rate (GFR), a surrogate of renal function, has been identified as a strong independent predictor of adverse outcomes in cardiovascular disease, including in the setting of STEMI [8,9,10]. Large-scale epidemiological studies have demonstrated a graded, inverse relationship between baseline filtration capacity and cardiovascular risk, wherein even mild-to-moderate chronic kidney disease escalates the risks of recurrent ischemic events, heart failure hospitalization, and death [11]. The estimation of GFR using the 2021 Chronic Kidney Disease Epidemiology Collaboration (CKD-EPI) [12] equation provides a more accurate assessment of renal function compared to earlier methods, particularly in patients with preserved kidney function. Impaired GFR, whether due to chronic kidney disease (CKD) or acute deterioration in GFR (AKI), has been associated with increased risk of major adverse cardiac events and in-hospital death [5,7,13,14].

GFR on admission is vital in decisions regarding pharmacological therapy [15] (such as intravenous crystalloids and angiotensin-converting enzyme (ACE) inhibitors) as well as judicious contrast media administration in PCI [16,17,18,19].

Patients presenting with renal impairment display distinct coronary plaque characteristics, including accelerated vascular calcification and more proximal culprit lesion localization, predisposing them to larger infarct sizes and refractory hemodynamic collapse [20]. However, differentiating the specific prognostic contribution of admission renal impairment from superimposed acute insults remains challenging due to frequent collinearity and variable follow-up durations across published cohorts [13,21,22]. Given these findings, this study aimed to evaluate the prognostic significance of admission eGFR, as calculated by the CKD-EPI equation, in predicting mortality among patients hospitalized with STEMI undergoing PCI, in the context of admission AKI.

## 2. Materials and Methods

### 2.1. Study Design and Population

This retrospective, single-center observational study initially screened n = 2949 consecutive adult patients admitted to the Cardiac Intensive Care Unit (CICU) of the Tel-Aviv Sourasky University Medical Center with a confirmed diagnosis of acute ST-segment elevation myocardial infarction (STEMI) between 1 January 2008 and 31 December 2019. The institutional cardiac care registry captures all consecutive acute coronary syndrome admissions treated in the dedicated CICU, ensuring minimal selection bias during the screening phase.

Patients were excluded if they were treated conservatively or with thrombolysis (n = 14), had a final discharge diagnosis other than STEMI (e.g., NSTEMI, myocarditis, MINOCA (MI with no obstructive coronary artery disease); n = 21) or died within 24 h of admission (n = 11). Patients dying within the initial 24 h were excluded to minimize confounding by immediate, irreversible cardiogenic shock or catastrophic mechanical complications that preclude meaningful longitudinal renal evaluation. The final patient cohort comprised 2903 patients.

#### Data Collection and Definitions

STEMI was diagnosed according to established European Society of Cardiology (ESC) criteria, including clinical (e.g., onset of typical chest pain), diagnostic electrographic ST elevations on two contiguous leads, and cardiac biomarkers rise on serial measurements. Demographic data, pre-existing comorbidities (hypertension, diabetes mellitus, hyperlipidemia, family history of premature CAD, previous myocardial infarction, prior percutaneous or surgical revascularization, and active smoking status), and baseline hemodynamics (heart rate, systolic and diastolic blood pressure) were systematically extracted from electronic medical records.

Primary PCI was performed in patients presenting within 12 h of symptom onset or within 12–24 h if symptoms persisted at presentation. All patients included in the analysis underwent primary PCI, with median door-to-balloon of 91 min. Reperfusion time was defined as the interval between symptom onset and the achievement of TIMI (Thrombolysis In Myocardial Infarction) grade 3 flow in the culprit artery, as documented in the catheterization report. Standard post-PCI medical therapy, consisting of dual antiplatelet therapy (aspirin and a P2Y12 inhibitor), statins, beta-blockers, and angiotensin-converting enzyme inhibitors or angiotensin receptor blockers, was initiated during hospitalization according to contemporary clinical guidelines unless contraindicated.

Left ventricular ejection fraction (LVEF) was assessed by bedside echocardiography within 48 h of admission using the biplane Simpson’s method or visual estimation by experienced cardiologists. Mortality was determined using records from the governmental resident register, encompassing the Israeli population. Mortality was assessed through September 30, 2021, providing a median follow-up of 2622 days.

### 2.2. Renal Function Assessment

Serum creatinine was measured at hospital admission (prior to PCI—defined as admission creatinine), at least once daily during the CICU stay, and throughout hospitalization. Presentation renal function was defined by the admission creatinine value. The estimated glomerular filtration rate (eGFR) was calculated using the Chronic Kidney Disease Epidemiology Collaboration [12] 2021 formula, with admission eGFR calculated using admission creatinine. Renal insufficiency was defined as an admission eGFR ≤60 mL/min/1.73 m^2^ (G3 or worse). EGFR was stratified to both grades (G1–G5: G1 ≥ 90, G2 60–89, G3 30–59, G4 15–29, G5 < 15 mL/min/1.73 m^2^) and quintiles (Q1(highest)–Q5), and primary analyses were carried out on both G1–G5 and Q1–Q5. Admission AKI was assessed upon acute presentation according to Kidney Disease: Improving Global Outcomes (KDIGO) [23] consensus criteria. All patients in the study cohort had available baseline serum creatinine measurements recorded during the preceding year across regional linked community electronic medical records. In accordance with KDIGO thresholds, admission AKI was adjudicated by comparing the initial hospital presentation creatinine against this documented pre-admission historical baseline, defined by an absolute increase of ≥ 0.3 mg/dL (≥ 26.5 micromol/L) or an elevation to ≥ 1.5 times the pre-admission baseline value. Because hourly urine-output measurements were not uniformly captured across the historical registry, AKI adjudication was based strictly on serum creatinine criteria.

### 2.3. Statistical Analyses

Descriptive statistics were carried out in order to summarize the baseline clinical and demographic characteristics of the study population. Continuous variables such as age, LVEF and laboratory values were reported as means ± standard deviations or medians with interquartile ranges, depending on distributional normality verified by the Kolmogorov–Smirnov test. Categorical variables, including gender, presence of cardiovascular risk factors (such as diabetes mellitus (DM), hyperlipidemia, family history, smoking status, personal history of myocardial infarction (MI), and incidence of acute kidney injury), were summarized using frequencies and percentages.

Comparative analyses were conducted to evaluate differences in clinical and demographic characteristics across estimated glomerular filtration rate quintiles. For categorical variables, chi-square tests were used to assess statistical significance, while one-way ANOVA with Bonferroni correction for multiple comparisons was applied to continuous variables.

The primary study endpoint was all-cause one-year mortality, evaluated via multivariable binary logistic regression among complete cases (n = 2870). Demographic, clinical, and angiographic covariates with univariable *p* < 0.10, alongside established cardiovascular prognostic factors, were entered into the model. Model discrimination was quantified by the area under the receiver operating characteristic curve (C-statistic) with 95% confidence intervals, model calibration was assessed via the Hosmer–Lemeshow goodness-of-fit test, and multicollinearity was examined using variance inflation factors (VIF). Several prespecified sensitivity analyses were pre-planned to evaluate model robustness: (1) evaluating admission eGFR as a continuous variable; (2) assessing a formal interaction term between admission eGFR quintiles and admission AKI; (3) re-incorporating the n = 11 early excluded deaths (<24 h) into the multivariable model; and (4) incorporating admission calendar year as a continuous covariate to evaluate potential era effects across the 12-year recruitment span. Secondary endpoints included crude 30-day all-cause mortality and longitudinal survival over extended follow-up among patients with complete continuous survival-time date tracking (n = 2425), evaluated using Kaplan–Meier survival curves and log-rank (Mantel–Cox) testing.

All statistical analyses were performed using IBM SPSS Statistics version 29 (IBM, Armonk, NY, USA). A *p*-value of < 0.05 was considered statistically significant in all analyses.

## 3. Results

### 3.1. Baseline Demographic and Clinical Characteristics

A total of n = 2903 participants were included in the primary analysis. Baseline demographic and clinical characteristics of the cohort stratified by eGFR quintiles—Q1 (>93.33 mL/min/1.73 m^2^, n = 584), Q2 (80.46–93.33 mL/min/1.73 m^2^, n = 579), Q3 (70.37–<80.46 mL/min/1.73 m^2^, n = 581), Q4 (57.80–<70.37 mL/min/1.73 m^2^, n = 578), and Q5 (<57.80 mL/min/1.73 m^2^, n = 581)—are shown in Table 1.

Patients in the lowest eGFR quintile (Q5) were substantially older (mean age 76.0 ± 10.6 vs. 50.9 ± 10.0 years in the highest eGFR quintile (Q1), *p* < 0.001) and had a higher prevalence of baseline cardiovascular morbidity. The proportion of female patients exhibited a U-shaped distribution across quintiles, being highest in Q5 (23.9%, n = 139) and Q1 (23.8%, n = 139), and lowest in Q3 (12.4%, n = 72) (*p* < 0.001 for trend across quintiles). The prevalence of diabetes mellitus increased progressively with worsening renal function, rising from 20.0% (n = 117) in Q1 to 33.2% (n = 193) in Q5 (*p* < 0.001). Similarly, hypertension demonstrated a steep, stepwise increase across declining eGFR quintiles: 28.9% (n = 169) in Q1, 39.0% (n = 226) in Q2, 38.6% (n = 224) in Q3, 50.7% (n = 293) in Q4, and 68.0% (n = 395) in Q5 (*p* < 0.001). Hyperlipidemia was also significantly more prevalent in lower eGFR tiers (54.9%, n = 319 in Q5 vs. 45.0%, n = 263 in Q1, *p* = 0.011). Conversely, active smoking was significantly more frequent among patients with higher baseline eGFR (63.8%, n = 367 in Q1 vs. 30.7%, n = 178 in Q5, *p* < 0.001), reflecting the younger age of patients in the higher filtration tiers, as was a positive family history of premature CAD (36.3%, n = 212 in Q1 vs. 6.6%, n = 38 in Q5, *p* < 0.001). A history of prior myocardial infarction was documented in 12.7% (n = 74) of Q1, 13.8% (n = 80) of Q2, 15.3% (n = 89) of Q3, 15.6% (n = 90) of Q4, and 20.8% (n = 121) of Q5 patients (*p* = 0.002). Mean LVEF increased with higher eGFR (47.9 ± 7.4% in Q1 vs. 45.1 ± 8.9% in Q5, *p* < 0.001) (47.8 ± 8.0% in Q2, 48.1 ± 7.5% in Q3, and 47.3 ± 8.6% in Q4). The proportion of patients presenting with AKI on admission increased steeply across quintiles, from 2.7% (n = 16) in Q1, 4.3% (n = 25) in Q2, 4.6% (n = 27) in Q3, 8.0% (n = 46) in Q4, to 23.1% (n = 134) in Q5, meaning that patients with reduced kidney function (Q5, <57.80 mL/min/1.73 m^2^) were also substantially more prone to additional kidney injury on admission.

### 3.2. Mortality Outcomes and eGFR Gradients

**Figure 1:**
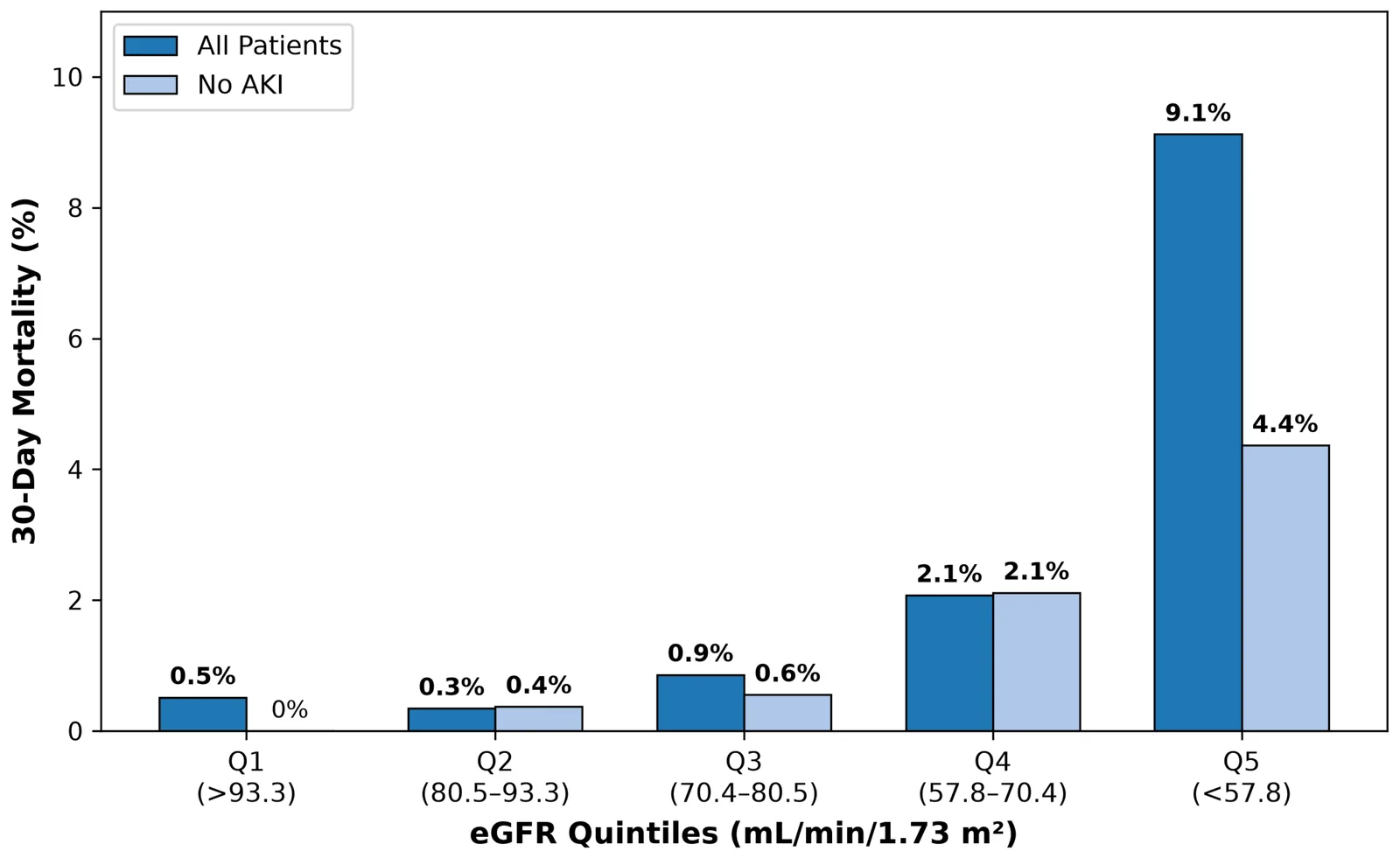
Crude 30-day mortality rates across admission eGFR quintiles stratified by admission AKI status (All patients vs. No AKI subset). Numbers denote percentage mortality. *p*-values reflect pairwise comparisons against the reference lowest quintile Q5 with Bonferroni adjustment.

Figure 1 illustrates crude 30-day mortality across eGFR quintiles in the overall cohort and stratified by admission AKI status. In the overall cohort, crude 30-day mortality declined markedly from 9.1% in the lowest eGFR quintile (Q5) to 2.1% in Q4, 0.9% in Q3, 0.3% in Q2, and 0.5% in Q1 (*p* < 0.001 for all pairwise comparisons vs. Q5).

Among patients presenting without admission AKI, a similar, though attenuated, gradient was observed: 30-day mortality was 4.4% in Q5 versus 2.1% in Q4, 0.6% in Q3, 0.4% in Q2, and 0.0% in Q1 (*p* < 0.001 for Q1 vs. Q5, *p* < 0.001 for Q2 vs. Q5, *p* = 0.004 for Q3 vs. Q5, and non-significant for Q4 vs. Q5). Crude one-year mortality demonstrated a consistent stepwise increase across worsening admission eGFR tiers (1.0% in Q1, 1.1% in Q2, 1.3% in Q3, 3.7% in Q4, and 14.5% in Q5, *p* < 0.001).

### 3.3. Kaplan–Meier Survival Analysis

**Figure 2:**
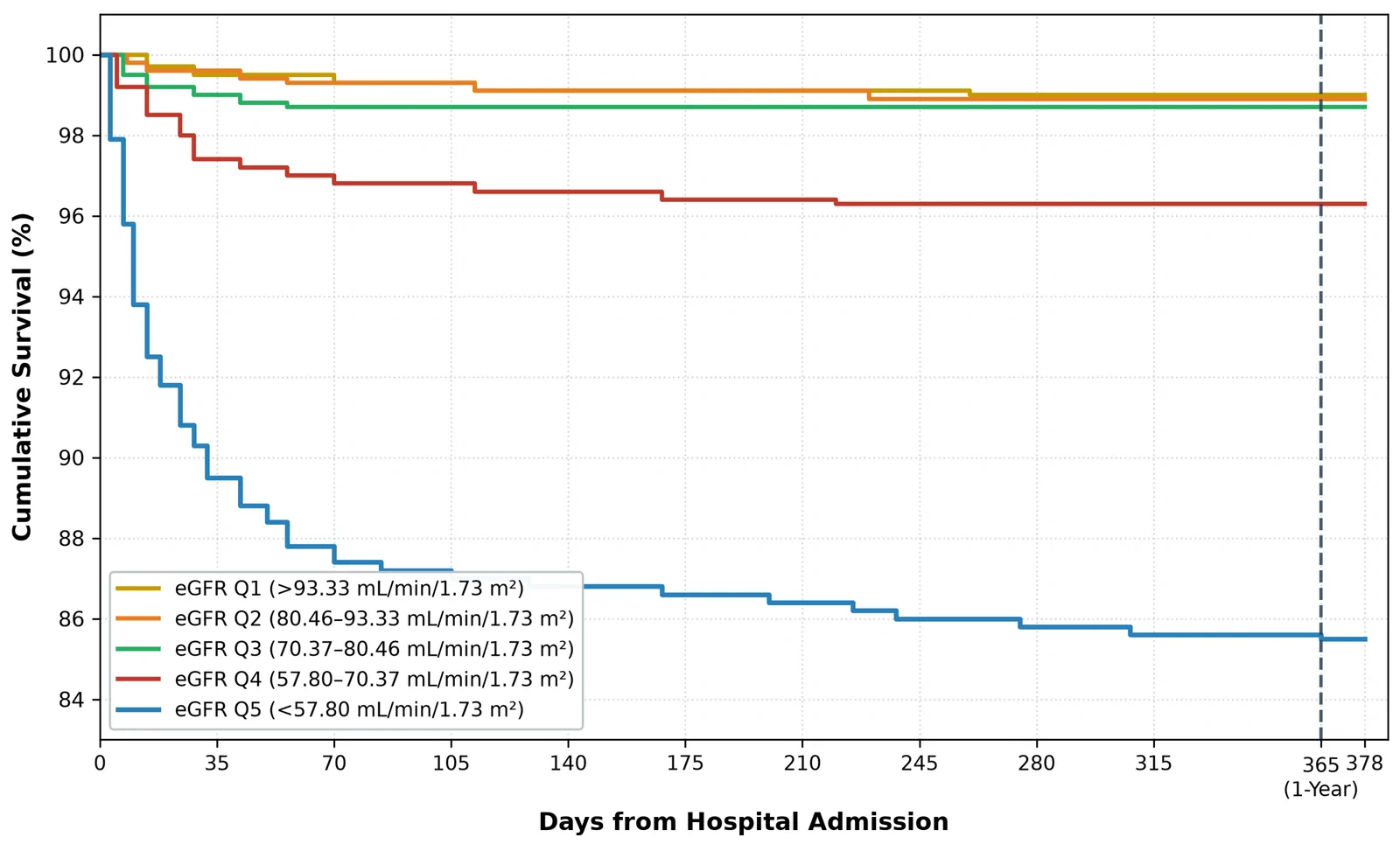
One-year Kaplan–Meier cumulative survival function curves across admission eGFR quintiles (n = 2425). Log-rank (Mantel–Cox) test chi-square = 193.5, *p* < 0.001.

One-year Kaplan–Meier survival function curves across eGFR quintiles are depicted in Figure 2 (n = 2425 patients with complete survival time records). Survival trajectories diverged rapidly following STEMI onset, with Q5 (eGFR < 57.80 mL/min/1.73 m^2^; blue curve) demonstrating an early, steep decline in cumulative survival within the initial 14 to 28 days that separated significantly from the remaining quintiles (log-rank chi-square = 193.5, *p* < 0.001). By 365 days (one year) of follow-up, cumulative survival remained highest in Q1 (>93.33 mL/min/1.73 m2, approximately 99.0%), Q2 (80.46 to 93.33 mL/min/1.73 m^2^, approximately 98.9%), and Q3 (70.37 to <80.46 mL/min/1.73 m^2^, approximately 98.7%), with an intermediate decrement observed in Q4 (57.80 to <70.37 mL/min/1.73 m^2^, approximately 96.3%), and the lowest survival in Q5 (<57.80 mL/min/1.73 m^2^, approximately 85.5%). While survival data were collected through September 30, 2021 (median follow-up of 2622 days), most mortality was accrued over the first year after STEMI, and the survival gap remained consistent throughout the follow-up period. Owing to low mortality events in higher filtration tiers, survival analysis was underpowered to identify significant survival differences among Q1–Q4.

### 3.4. Multivariable Predictors of One-Year Mortality

A multivariable binary logistic regression model evaluating independent predictors of 1-year mortality (n = 2870) is presented in Table 2. Declining renal function at baseline was associated with higher 1-year mortality, with the overall effect of baseline eGFR quintiles on 1-year mortality reaching statistical significance (global Wald test *p* = 0.047). In pairwise comparisons using Q1 (>93.33 mL/min/1.73 m^2^) as the reference category, the adjusted odds ratios for 1-year mortality were 0.72 (95% CI 0.20 to 2.56, *p* = 0.612) for Q2 (80.46 to ≤93.33 mL/min/1.73 m^2^), 0.67 (95% CI 0.20 to 2.30, *p* = 0.526) for Q3 (70.37 to <80.46 mL/min/1.73 m^2^), 1.28 (95% CI 0.43 to 3.79, *p* = 0.654) for Q4 (57.80 to <70.37 mL/min/1.73 m^2^), and 2.25 (95% CI 0.74 to 6.84, *p* = 0.153) for Q5 (<57.80 mL/min/1.73 m^2^).

In the fully adjusted multivariable model, each additional year of age and the presence of admission AKI were the dominant independent predictors of 1-year death (age: adjusted OR 1.05 per 1-year increment, 95% CI 1.02 to 1.08, *p* < 0.001; admission AKI vs. no AKI: adjusted OR 7.07, 95% CI 4.48 to 11.14, *p* < 0.001). While eGFR quintiles demonstrated an overall statistically significant association with one-year mortality (global Wald test *p* = 0.047), individual quintile odds ratios were attenuated following adjustment for age and AKI. Other clinical covariates did not achieve independent significance: male gender (adjusted OR 0.67, 95% CI 0.41 to 1.10, *p* = 0.110), hypertension (adjusted OR 1.16, 95% CI 0.70 to 1.92, *p* = 0.574), diabetes mellitus (adjusted OR 0.67, 95% CI 0.42 to 1.09, *p* = 0.104), hyperlipidemia (adjusted OR 1.47, 95% CI 0.92 to 2.36, *p* = 0.106), family history of CAD (adjusted OR 2.28, 95% CI 0.80 to 6.50, *p* = 0.123), active smoking (adjusted OR 1.18, 95% CI 0.72 to 1.94, *p* = 0.516), and prior myocardial infarction (adjusted OR 0.80, 95% CI 0.47 to 1.37, *p* = 0.412). The multivariable model exhibited excellent discrimination (C-statistic = 0.841, 95% CI 0.812 to 0.870) and calibration (Hosmer–Lemeshow chi-square = 7.82, df = 8, *p* = 0.451), with no evidence of collinearity (maximum VIF = 1.38 for age; all other VIFs < 1.30). A formal interaction term between admission eGFR quintiles and admission AKI was not statistically significant (likelihood ratio test chi-square = 5.08, df = 4, *p* = 0.28).

In sensitivity analysis re-incorporating the 11 early excluded deaths (<24 h) into the multivariable model, parameter estimates remained essentially unchanged (adjusted OR for age 1.05, *p* < 0.001; admission AKI 7.04 vs. 7.07, *p* < 0.001; eGFR global *p* = 0.049), confirming that survivor bias did not alter the conclusions.

In sensitivity analysis incorporating admission calendar year as a continuous variable, each later year was associated with reduced mortality (adjusted OR 0.91 per year, 95% CI 0.86 to 0.97, *p* = 0.002); however, age (adjusted OR 1.05, *p* < 0.001) and admission AKI (adjusted OR 4.10, *p* < 0.001) remained the dominant predictors, and eGFR Q5 remained stable (adjusted OR 2.06, *p* = 0.12).

When admission eGFR was modelled as a continuous variable, each 10 mL/min/1.73 m^2^ decrement was associated with an adjusted OR of 1.08 (95% CI 0.99 to 1.18, *p* = 0.082). Finally, sensitivity analysis controlling for AKI across KDIGO eGFR stages (G1-G5) yielded consistent results as with Q1-Q5.

## 4. Discussion

In the present study, we evaluated the prognostic significance of admission estimated glomerular filtration rate (eGFR) using the 2021 CKD-EPI equation in a large cohort of ST-segment elevation myocardial infarction (STEMI) patients undergoing primary percutaneous coronary intervention (PCI). Applying the race-free 2021 CKD-EPI equation across a large, consecutive primary PCI cohort allows granular examination of how admission renal filtration interacts with acute kidney injury. Our findings demonstrate that admission eGFR serves primarily as an indicator of baseline cardiorenal reserve, reflecting patient vulnerability in conjunction with advancing age and acute cardiorenal insults. While admission eGFR was significantly associated with one-year mortality in global testing (*p* = 0.047), individual quintile associations were attenuated after adjusting for age and admission AKI, demonstrating that admission renal function reflects overall cardiovascular risk rather than an isolated risk driver. Over an extended median follow-up of 2622 days, admission renal impairment remained associated with long-term all-cause mortality in secondary unadjusted analysis, reinforcing the consensus that impaired kidney function is an essential marker of cardiovascular risk [5,7].

The inverse relationship between renal function and mortality in the setting of acute coronary syndromes (ACS) is well-documented [10,13,21]. Our results align with landmark trials such as the VALIANT study, which showed that even mild-to-moderate renal dysfunction (eGFR < 75 mL/min/1.73 m^2^) was associated with increased risks of all-cause mortality and cardiovascular events following myocardial infarction [7]. A subsequent pooled analysis of four randomized trials involving over 27,000 high-risk patients confirmed that each 10 mL/min reduction in eGFR below 75 was associated with a 13% increase in the hazard for death [24]. Similarly, renal sub-analyses of the PLATO and TRITON-TIMI 38 trials have consistently demonstrated that patients with impaired renal function experience higher rates of major adverse cardiovascular events (MACE) and mortality [25,26]. Broad collaborative meta-analyses encompassing general and high-risk cohorts have established a universal, non-linear risk curve where cardiovascular mortality climbs steeply once filtration drops below 60 mL/min/1.73 m^2^ [27,28]. Community-based investigations similarly confirm this exponential risk escalation [11]. In contemporary primary PCI registries, our findings mirror those of Campbell et al. [22] and Gao et al. [29], reaffirming that admission eGFR establishes a long-term prognostic trajectory [30].

An important finding of our study is the prognostic relevance of admission eGFR alongside AKI. While AKI is a known powerful predictor of STEMI mortality [3,4] and represents an acute cardiorenal insult associated with severe in-hospital mortality [2], our findings suggest that reduced admission filtration itself reflects a vulnerable physiological state. This is consistent with evidence from other cohorts where reduced filtration capacity was associated with higher mortality after emergency PCI [22,29,31]. Recent studies utilizing the CKD-EPI equation, such as the analysis by Sadre-Bafghi et al. [32], have also underscored the prognostic impact of initial eGFR levels, suggesting that presentation renal status provides a critical window into the patient’s overall cardiovascular resilience. Because admission eGFR demonstrated a graded mortality risk among patients presenting without admission AKI, the observed risk is not driven solely by acute hemodynamic insults sustained during presentation [16,17,18].

Several mechanisms may explain why admission renal impairment, even in the absence of admission AKI, is associated with a higher risk of death. Patients with lower eGFR often present with a more advanced burden of atherosclerotic risk factors, including hypertension and diabetes, as seen in our cohort. Moreover, chronic kidney disease is associated with a systemic pro-inflammatory and pro-thrombotic state, accelerated vascular calcification, and subclinical myocardial dysfunction, all of which may exacerbate the consequences of an ischemic insult [6,7]. Impaired renal clearance leads to altered mineral metabolism, hyperphosphatemia, and medial sclerosis, resulting in heightened arterial stiffness and diffuse coronary calcification [27]. In patients undergoing primary PCI, admission renal impairment is strongly correlated with complex multivessel coronary disease, lower rates of TIMI 3 flow, and reduced myocardial salvage [33].

Renal impairment is associated with a systemic pro-inflammatory and pro-thrombotic state. The accumulation of circulating uremic retention solutes promotes endothelial activation, elevated oxidative stress, and heightened platelet reactivity [30]. This prothrombotic milieu blunts the response to standard antiplatelet therapy and impairs endogenous fibrinolysis, predisposing to recurrent ischemic events and stent failure [25,26].

Furthermore, while prior registry literature has described “renalism” (the therapeutic undertreatment of renal patients due to safety concerns), our cohort was treated in a tertiary cardiac center with universal primary PCI (median door-to-balloon 91 min) and standardized guideline-directed post-PCI medical therapy. Thus, the observed excess mortality in lower eGFR tiers reflects intrinsic cardiovascular fragility and acute hemodynamic vulnerability rather than systemic therapeutic omission [5]. Registry data consistently demonstrate that STEMI patients with baseline renal dysfunction are less frequently prescribed guideline-directed medical therapies and encounter delays or conservatism in invasive revascularization [15,30,34]. Across the 12-year registry recruitment span (2008–2019), evolving interventional techniques and post-PCI medical regimens yielded progressive survival gains (adjusted OR 0.91 per calendar year, 95% CI 0.86 to 0.97, *p* = 0.002). Nevertheless, multivariable sensitivity modeling incorporating admission year confirmed that the prognostic dominance of advancing age and admission AKI remained unaltered.

However, our data suggest that clinicians should also pay close attention to the admission eGFR as a tool for long-term risk stratification. The observation that mortality gradients persist even in patients who do not develop AKI underscores the need for aggressive secondary prevention and long-term monitoring in STEMI survivors with admission renal insufficiency, warranting structured, multidisciplinary cardiorenal surveillance protocols following hospital discharge.

## 5. Limitations

Our study has several limitations. First, as a retrospective single-center study, our findings may not be fully generalizable to other populations or clinical settings. However, the single-center setting provided high data integrity, uniform treatment protocols, and centralized laboratory monitoring.

Second, we utilized the 2021 CKD-EPI equation, which excludes race as a variable; while this improves clinical applicability in diverse populations, it represents a change from older models used in historical trials [12]. Third, our renal function assessment was based on admission creatinine, which reflects presentation renal function rather than chronic pre-morbid outpatient baseline status. In the acute setting of STEMI, early hemodynamic instability, acute neurohormonal activation, or dehydration may alter admission creatinine levels before chronic renal status can be confirmed.

Fourth, unmeasured residual confounders, such as pre-admission baseline albuminuria/proteinuria measurements, novel tubular biomarker levels, and long-term out-of-hospital medication adherence, were not fully available across the historical cohort. Procedural parameters such as total iodinated contrast volume were documented in a minority of registry records (14.1%) and were excluded from multivariable regression to avoid substantial listwise deletion. In addition, detailed hemodynamic and angiographic indices (including Killip class at presentation, TIMI flow grade, and coronary vessel disease counts) had substantial missingness across the 12-year registry and could not be incorporated into multivariable models without excessive case attrition. Furthermore, vital status tracking beyond 365 days was complete in 83.5% (n = 2425) of patients; those with complete continuous date records exhibited baseline characteristics comparable to omitted cases (mean age 60.9 vs. 61.2 years, *p* = 0.62; admission eGFR 75.4 vs. 74.8 mL/min/1.73 m^2^, *p* = 0.55), confirming no systematic attrition bias.

Finally, the governmental population registry provided robust long-term tracking of all-cause mortality, but cause-specific mortality (e.g., distinguishing cardiovascular death from sudden arrhythmic death or end-stage non-cardiovascular causes) could not be adjudicated.

## 6. Conclusions

Admission eGFR, calculated using the 2021 CKD-EPI equation, is significantly associated with one-year mortality following STEMI in global testing, although its independent predictive effect is attenuated following adjustment for advancing age and admission acute kidney injury, which serve as the primary determinants of one-year death. Rather than acting as a strong isolated independent predictor, admission renal function primarily reflects baseline cardiorenal reserve and general physiological vulnerability. Because increased mortality was observed across lower eGFR tiers even among patients presenting without admission acute kidney injury, admission renal impairment should be recognized as an important marker of longitudinal cardiorenal risk. Integrating routine admission renal status into standard acute risk-stratification models could enhance early clinical decision-making, optimize secondary preventive pharmacotherapy, and guide post-discharge multidisciplinary cardiorenal surveillance strategies for high-risk STEMI survivors.

## Figures and Tables

**Table 1:** Demographic characteristics, cardiovascular risk factors, LVEF, and admission AKI across eGFR quintiles. Values are mean ± SD or n (%). *p*-values derived from one-way ANOVA for continuous variables and Pearson chi-square for categorical variables. AKI, acute kidney injury; CAD, coronary artery disease; eGFR, estimated glomerular filtration rate; LVEF, left ventricular ejection fraction; MI, myocardial infarction.

Characteristic	Q1 (>93.33) (n = 584)	Q2 (80.46–93.33) (n = 579)	Q3 (70.37–<80.46) (n = 581)	Q4 (57.80–<70.37) (n = 578)	Q5 (<57.80) (n = 581)	*p*-Value
Age (years), Mean ± SD	50.9 ± 10.0	55.9 ± 9.4	60.3 ± 9.2	65.8 ± 9.4	76.0 ± 10.6	<0.001
Male, n (%)	445 (76.2%)	492 (85.0%)	508 (87.6%)	483 (83.6%)	442 (76.1%)	<0.001
Female, n (%)	139 (23.8%)	87 (15.0%)	72 (12.4%)	95 (16.4%)	139 (23.9%)	<0.001
Diabetes Mellitus, n (%)	117 (20.0%)	120 (20.7%)	111 (19.1%)	149 (25.8%)	193 (33.2%)	<0.001
Hypertension, n (%)	169 (28.9%)	226 (39.0%)	224 (38.6%)	293 (50.7%)	395 (68.0%)	<0.001
Hyperlipidemia, n (%)	263 (45.0%)	275 (47.5%)	298 (51.3%)	284 (49.1%)	319 (54.9%)	0.011
CAD Family History, n (%)	212 (36.3%)	164 (28.3%)	128 (22.1%)	90 (15.6%)	38 (6.6%)	<0.001
Current Smoker, n (%)	367 (63.8%)	355 (62.0%)	282 (49.1%)	264 (46.0%)	178 (30.7%)	<0.001
Prior MI, n (%)	74 (12.7%)	80 (13.8%)	89 (15.3%)	90 (15.6%)	121 (20.8%)	0.002
LVEF (%), Mean ± SD	47.9 ± 7.4 (n = 494)	47.8 ± 8.0 (n = 523)	48.1 ± 7.5 (n = 534)	47.3 ± 8.6 (n = 535)	45.1 ± 8.9 (n = 543)	<0.001
Admission AKI, n (%)	16 (2.7%)	25 (4.3%)	27 (4.6%)	46 (8.0%)	134 (23.1%)	<0.001

**Table 2:** Multivariable binary logistic regression for 1-year all-cause mortality (n = 2870 complete cases, 170 deaths). Model C-statistic = 0.841 (95% CI 0.812–0.870); Hosmer–Lemeshow chi-square = 7.82, df = 8, *p* = 0.451; Maximum VIF = 1.38.

Predictor Covariate	Adjusted Odds Ratio (95% CI)	*p*-Value
Age, per 1-year increment	1.05 (1.02–1.08)	<0.001
Male vs. female	0.67 (0.41–1.10)	0.110
Admission AKI vs. no AKI	7.07 (4.48–11.14)	<0.001
eGFR quintiles (overall)		0.047
Q2 (80.46–93.33) vs. Q1 (Reference)	0.72 (0.20–2.56)	0.612
Q3 (70.37–<80.46) vs. Q1	0.67 (0.20–2.30)	0.526
Q4 (57.80–<70.37) vs. Q1	1.28 (0.43–3.79)	0.654
Q5 (<57.80) vs. Q1	2.25 (0.74–6.84)	0.153
Hypertension (yes vs. no)	1.16 (0.70–1.92)	0.574
Diabetes mellitus (yes vs. no)	0.67 (0.42–1.09)	0.104
Hyperlipidemia (yes vs. no)	1.47 (0.92–2.36)	0.106
Family history of CAD	2.28 (0.80–6.50)	0.123
Active smoker	1.18 (0.72–1.94)	0.516
Prior myocardial infarction	0.80 (0.47–1.37)	0.412

## Data Availability

The data presented in this study are available on request from the corresponding author. The data underlying this article are not publicly available due to institutional and privacy restrictions.
